# Automatic Kidney Segmentation Method Based on an Enhanced Generative Adversarial Network

**DOI:** 10.3390/diagnostics13071358

**Published:** 2023-04-06

**Authors:** Tian Shan, Yuhan Ying, Guoli Song

**Affiliations:** 1 State Key Laboratory of Robotics, Shenyang Institute of Automation, Chinese Academy of Sciences, Shenyang 110016, China; 2 Institutes for Robotics and Intelligent Manufacturing, Chinese Academy of Sciences, Shenyang 110169, China; 3 University of Chinese Academy of Sciences, Beijing 100049, China

**Keywords:** kidney segmentation, generative adversarial networks, dense block

## Abstract

When deciding on a kidney tumor’s diagnosis and treatment, it is critical to take its morphometry into account. It is challenging to undertake a quantitative analysis of the association between kidney tumor morphology and clinical outcomes due to a paucity of data and the need for the time-consuming manual measurement of imaging variables. To address this issue, an autonomous kidney segmentation technique, namely SegTGAN, is proposed in this paper, which is based on a conventional generative adversarial network model. Its core framework includes a discriminator network with multi-scale feature extraction and a fully convolutional generator network made up of densely linked blocks. For qualitative and quantitative comparisons with the SegTGAN technique, the widely used and related medical image segmentation networks U-Net, FCN, and SegAN are used. The experimental results show that the Dice similarity coefficient (DSC), volumetric overlap error (VOE), accuracy (ACC), and average surface distance (ASD) of SegTGAN on the Kits19 dataset reach 92.28%, 16.17%, 97.28%, and 0.61 mm, respectively. SegTGAN outscores all the other neural networks, which indicates that our proposed model has the potential to improve the accuracy of CT-based kidney segmentation.

## 1. Introduction

One of the most common cancers of the urinary system is the renal tumor. The majority of these tumors are cancerous, and their occurrence rate is rising yearly. There were 208,000 diagnoses and 102,000 fatalities of kidney cancer in 2002 [1], while in 2018 there were more than 400,000 diagnoses and more than 175,000 deaths [2]. The incidence increases between the ages of 60 and 70 years, and it is greater in industrialized nations than in developing nations [3]. Spiral CT is frequently used for the clinical diagnosis of lesions that occupy renal space, and the scan time is fairly quick. The substantial enlargement of the kidney is the primary indicator of renal cell carcinoma in a CT imaging evaluation. The patient’s soft tissue is significantly thickened at the same time that the tumor develops and expands outward. Adipose tissue’s functionality is uncertain. The accuracy of kidney segmentation may need to be further enhanced because the quality of the segmentation results will affect the efficacy and side effects of radiation for kidney cancer.

In the past, segmenting renal CT data by hand required a skilled observer and was a laborious operation that took time. Different segmentation techniques for renal CT images have been developed to address this. Many researchers have suggested that by combining prior knowledge of human anatomy (such as the relative position or morphological characteristics of the kidney), and according to the method of kidney positioning first and then kidney segmentation, the degree of segmentation automation can be improved. Yan et al. segmented the kidney using an improved region growth algorithm based on multi-scale morphology and a labeling algorithm after using the spine as a marker and the connected region labeling algorithm based on image intensity to determine the kidney’s position [4]. With the connected region labeling technique, Abirami et al. also identified the location of the kidney by using the spine as a marker. Based on this, the kidney area was removed using the adaptive region growth method [5]. Anatomical details and morphology were suggested by Berlgherbi et al. as a way to identify and segment the kidney. The target area and mark are acquired after the spine has been removed using a threshold and other picture adjustments. According to the picture gradient and mark control watershed method, the kidney is segmented [6]. To achieve kidney segmentation and tissue classification, Khalifa et al. developed a random forest approach based on prior information concerning the kidney shape and high-order feature information [7]. A coarse to fine method for segmenting the kidney from a CT picture in two phases was proposed by Song et al. The approximate range of the kidney is first determined using the fuzzy c-means clustering algorithm based on spatial information, and then the improved use of the GrowCut algorithm results in fine segmentation [8]. There is a need for a complementary approach through the combination of various segmentation algorithms because a single segmentation method cannot satisfy the needs of CT image kidney segmentation. In order to reliably and quickly extract renal lesions from CT images, Kaur et al. introduced a hybrid segmentation approach that combines fuzzy c-means clustering based on spatial information with distance regularized level set evolution (DRLSE). Fuzzy c-means clustering using picture spatial information and hesitation is used to first obtain the target’s initial contour, and then a distance regularization level set technique is used to segment the focus [9]. To segment the kidney and renal cortex in an improved CT picture, Chen et al. utilized the Intelligent Scissors algorithm, image cutting algorithm, and other active appearance model methods [10]. A multimap technique integrating picture intensity and form constraints was presented by Kim. Segmenting the kidney from a CT scan, predicting renal function from the change in the renal volume, and helping the doctor come up with a treatment plan for patients who have undergone partial nephrectomy are all possible [11]. 

Deep convolutional neural networks (CNNs) have frequently been used to separate medical images in recent years [12,13,14,15]. For instance, the fully convolution network (FCN) proposed by Shelhamer et al. [16] is an end-to-end network that solves the issue of semantic segmentation by classifying images at the pixel level. Instead, Ronneberger et al. [17] used a U-Net network to segment medical images. A contracting path is utilized in U-Net to collect contextual data from images, while an expanding path is used to precisely pinpoint the segmented target. To the same end, V-Net is a three-dimensional (3D) end-to-end medical segmentation algorithm that was created by Milletari et al. [18]. This network uses a residual learning approach to speed up convergence and adds the Dice coefficient as a novel objective function. Among these, Pedraza et al. [19] used pretrained AlexNet to distinguish between glomerulus and non-glomerulus. A total of 10,600 region of interest (ROI) photos from 40 whole slide images were used in the investigation. Moreover, 244 CT images of people with Autosomal Dominant Polycystic Kidney Disease (ADPKD) were used in an investigation by Sharma et al. [20]. On slice-wise axial CT slices, they trained a fully convolutional network for segmentation. Using multi-channel FCN on CT images, where the feature vector was produced by the fusing of features from various channels, Sun et al. [21] and Ruan et al. [22] combined a multi-scale feature extractor and a finder of the area of interest with GAN. Furthermore, Sandfort et al. [23] and Conze et al. [24] employed GAN to automatically segment numerous organs in abdominal CT and MR images. This allowed for the efficient simultaneous segmentation of many organs. The prostate, a male-specific reproductive organ located in the pelvic cavity, was segmented by Wang et al. [25] using GAN. Based on [26], Yuan et al. [27] achieved 3D image segmentation of abdominal organs and brain tumors, thereby breaking the 2D segmentation constraint. In summary, the pros and cons of the above representative methods have been listed in Table 1. On the public kidney data set Kits19, these approaches have so far undergone testing and performed well. However, renal segmentation is made more challenging by the intricacy of renal CT, particularly in the center of the left and right kidneys, where there is a small collecting system that does not belong to the kidney and no useful edge information. As a result, we anticipate that the developed algorithm will be able to pick up more image features and perform well when segmenting distinct slices of a kidney CT image.

As a new end-to-end architecture for segmenting the kidney region, we suggest the SegTGAN technique in this study, which draws inspiration from the SegAN model [28]. The generational adversarial network (GAN) has decent generating abilities and can partially capture data distribution. In this study, we adjusted the GAN network structure and optimized the goal function to increase the kidney segmentation accuracy. The following are the specific contributions of this work:

- The network used to generate the segmentation result images in the generator network is an end-to-end complete convolutional network with a U-Net-like topology.
- To make dense connections, we decided to employ densely connected blocks between the posterior layers and all the anterior layers, which ease the gradient vanishing problem, improve feature propagation, and significantly reduce the number of parameters. By connecting the features in the channel dimension, they enable feature reuse.
- To avoid model overfitting and more reliably guarantee sparsity, multi-scale feature connections are created in discriminator networks, and the L1 parametric form of the mean absolute error is included as a regular term to the objective function.

## 2. Materials and Methods

**Figure 1:**
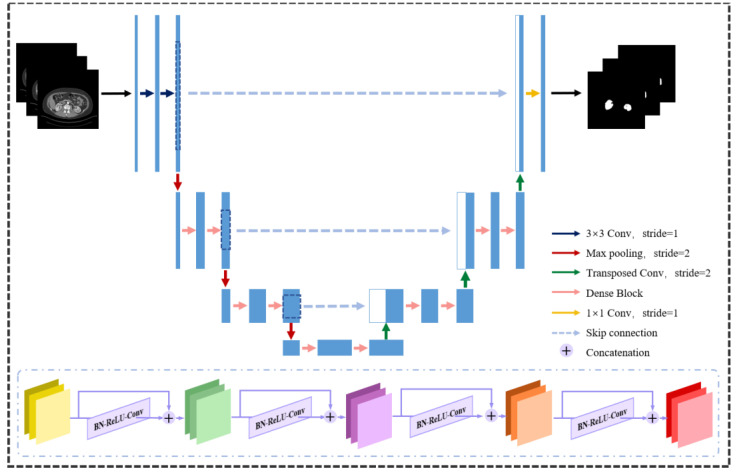
The structure of the generator network model. The structure of the dense block is shown in the box below.

**Figure 2:**
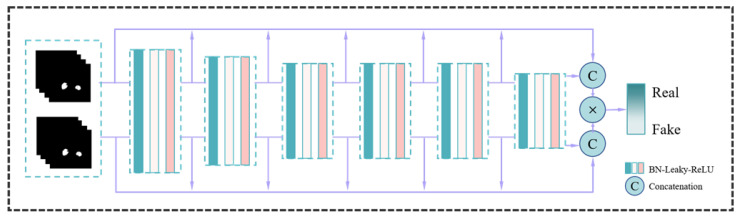
The structure of the discriminator network. The output is real or fake.

**Figure 3:**
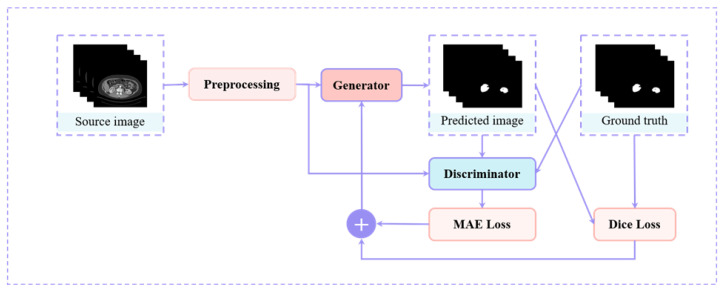
Schematic of SegTGAN’s overall flow structure. Mean absolute error loss and generative adversarial network for kidney segmentation.

A generator G network and a discriminator D network make up our segmentation method, SegTGAN. The generator is made to pick up on the actual data distribution and produce kidney-region images that are comparable to it. The discriminator generates discriminating results by separating the images generated by the generator from the images that represent the ground truth. In order to bring the two networks into conflict, the discrimination results are sent back to the generator. As a result, the images produced by the generator are closer to the real-world images. Figure 1, Figure 2 and Figure 3 depict the network structure.

### 2.1. SegTGAN Architecture

#### 2.1.1. Generator

As seen in Figure 1, the generator G is an end-to-end segmentation network. The overall structure of G is based on the encoder-decoder structure of the U-Net [17], which is based on a fully convolutional network [16]. Both up-sampling and down-sampling techniques are used in this network. Three maximum pooling layers, three densely connected blocks, and a convolutional layer with 3 × 3 convolution kernels make up the down-sampling process. Three deconvolution layers, three densely connected blocks, and a 1 × 1 convolution kernel are all included in the up-sampling process. Skip connections between the two are added, making the network comparable to an autoencoder. With this design, it is possible to extract picture features at various sizes during down-sampling and to provide a view at the same size as the input image during up-sampling, which is comparable to reconstructing the output at the same size as the input. It can also learn potential representations.

In order to achieve feature reuse by connecting the features in the channel dimension, alleviate the gradient disappearance problem, and enhance the network performance, a dense block structure is introduced into the generator network. This structure establishes dense connections between the back layer and all of the front layers. According to the dashed box in Figure 1, which depicts a structure with four layers, the underlying structure is comparable to that of DenseNet [29]. Batch normalization, a rectified linear unit, and a 3 × 3 convolution kernel are all included in each layer. 

#### 2.1.2. Discriminator

The multi-dimensional feature extraction network with six layers is called discriminator D. Convolutional, BN, and leaky ReLU activation layers are all included in each layer. The sizes of the convolutional kernels are 7 × 7, 5 × 5, 4 × 4, and 3 × 3. The discriminator D’s structural details and the elements of the convolutional layers are shown in Figure 2. 

#### 2.1.3. SegTGAN

The generator, which supplies the segmentation masks through the encoding and decoding layers, and the discriminator, which determines if a given segmentation mask is synthetic or genuine and then assesses it, make up the overall SegTGAN architecture (Figure 3). Therefore, in order to encourage the generator to produce segmentation masks that are as comparable as feasible, the adversarial network is trained to differentiate between actual and artificial signals.

### 2.2. Objective Function

The objective function of conventional GANs is defined as:

(1) $$\underset{G}{\min}\underset{D}{\max}V(G,D)=E_{x\sim p_{data}(x)}[\log D(x)]+E_{z\sim p_{z}(z)}[1-\log(D(G(x)))]$$

where x represents the actual data and $p_{data}(x)$ represents its probability distribution. The random noise distribution $p_{z}(z)$ is typically satisfied by the noise data z. D(x) represents the likelihood that the input image x originates from the training sample as opposed to the one produced by the generator. The generator’s differentiation function is indicated by the letter G(z).

The objective function of SegTGAN should comprise two elements, the first of which is the mapping term of the generator. This is in line with both the objective function of traditional GAN and our goal of applying a GAN network to kidney segmentation. The discriminator’s decision result term is the second term. The generator is used to binary mask image y from the original CT image x. For each data point, the discriminator D produces a binary image categorization ${\left\{0,1\right\}}^{k}$. In this classification, k denotes the number of decisions, 1 denotes that y is a ground-truth image from the training sample, and 0 indicates that y is a G-generated image. The Dice coefficient is a crucial indicator for assessing how well segmentation is working. We improved the goal function’s training outcomes by including a Dice coefficient control.

The L1 parametric loss, sometimes referred to as the mean absolute error (MAE) loss, determines the average of the total absolute discrepancies between the actual and predicted values. When there are outliers in the distribution of the target variable, the MAE loss is more resistant to them. Adding L1 regularization is equivalent to adding a priori knowledge to the model: the weights obey the zero-mean Laplace distribution. Moreover, L1 regularization makes the weights of the neural network as small as possible, convergent to zero, which is equivalent to reducing the complexity of the network and preventing overfitting. The model’s capacity to generalize is enhanced by the fact that it has a lower level of complexity and so is more robust to noise and outliers. Thus, the following is the final definition of objective function:

(2) $$\underset{G}{\min}\underset{D}{\max}V(G,D)=\frac{\lambda }{N}\sum_{i=1}^{N}E_{dice}(G(x_{i}),y_{i})+\frac{\delta }{N}\sum_{i=1}^{N}E_{mae}(f_{D}(x_{i},y_{i}),f_{D}(x_{i},G(x_{i})))$$

(3) $$E_{dice}(x_{i},y_{i})=-\frac{2\sum_{i=1}^{N}x_{i}y_{i}+\varepsilon }{\sum_{i=1}^{N}\left(x_{i}+y_{i}\right)+\varepsilon }$$

(4) $$E_{mae}(x_{i},y_{i})=\frac{1}{N}{\sum_{i=1}^{N}\left\|x_{i}-y_{i}\right\|}_{1}$$

where $x_{i}$ and $y_{i}$ refer to the input CT images and ground truth images, respectively, and N is the number of training images. The input data $x_{i}$ is utilized to extract the hierarchical features using the discriminator function $f_{D}$. The smoothing term, ε, ensures that the denominator is not zero. In addition, λ and δ are both adjusting variables to maximize the weight effect. 

### 2.3. Experimental Configuration and Evaluation Criteria

#### 2.3.1. Data

The 2019 Kidney Tumor Segmentation Challenge provided the public dataset Kits19, which is used in the model. The candidates for inclusion in this database were all patients who underwent partial or radical nephrectomy for one or more kidney tumors at the University of Minnesota Medical Center between 2010 and 2018. A total of 300 examples were chosen at random from the group. Medical students working under the direction of Dr. Christopher Weight, clinical chair, provided the manual segmentation labels. The data of 150 randomly chosen subjects serve as the neural network training set, the data of 60 subjects serve as the neural network validation set, and the remaining 90 subjects’ data serve as the neural network’s final test set. In preprocessing, a binning analysis is performed using 16 × 128 × 128 slices without pixel value normalization but using 12 × 32 × 32 overlapping slices for the full data enhancement. The original scanned picture resolution was 512 × 512. Blank facets are skipped during training, and overlapping facets are produced for the prediction.

#### 2.3.2. Implementation 

The segmentation models in this paper are programmed using Python, TensorFlow, and Keras. All the experiments are carried out on a personal workstation with a Nvidia GeForce RTX 3080 GPU, which has a learning rate of 0.0001, weight decay of 0.0001, and momentum of 0.9.

#### 2.3.3. Performance Metrics

As an evaluation criterion for the network segmentation performance, several metrics, including the Dice similarity coefficient (DSC), volumetric overlap error (VOE), average surface distance (ASD) [30], accuracy (ACC), sensitivity (SEN), and specificity (SPE), are introduced.

The following definition is used for the DSC, an ensemble similarity measure function that determines the contour similarity of a specific region in two images:

(5) $$DSC=\frac{2\left|A\cap B\right|}{\left|A\right|+\left|B\right|}=\frac{2\times TP}{2\times TP+FP+FN}$$

where *A* and *B*, respectively, represent the segmentation results and the ground truth.

The following is the approach used by the VOE to calculate the ratio between the intersection and joint points of two images:

(6) $$VOE(A,B)=(1-\frac{\left|A\cap B\right|}{\left|A\cup B\right|})\times 100\%$$

The average surface distance (ASD) between binary items in two pictures is calculated and defined as follows:

(7) $$ASD=\frac{1}{\left|S(A)+S(B)\right|}(\sum_{a\in S(A)}d(a,S(B))+\sum_{b\in S(B)}d(b,S(A)))$$

where S(A) and S(B) are the surface voxels of the segmentation results and ground truth masks, respectively. The value d(·) indicates the proximity of two images’ voxels by the shortest distance.

The accuracy (ACC), sensitivity (SEN), and specificity (SPE) are defined as follows:

(8) $$Accuracy=\frac{TP+TN}{TP+TN+FP+FN}$$

(9) $$Sensitivity=\frac{TP}{TP+FN}$$

(10) $$Specificity=\frac{TN}{TN+FP}$$

## 3. Results

**Figure 4:**
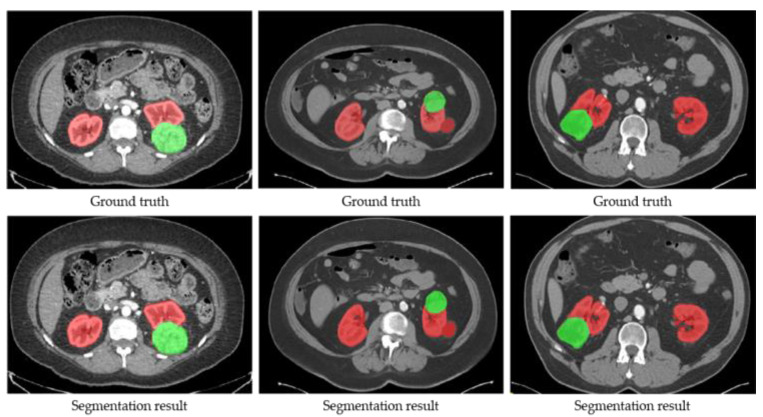
Examples of the segmentation results of SegTGAN on the Kits21 dataset. Red contour denotes the kidney and green contour denotes a tumor.

Firstly, the training and validation datasets are employed to update the weights and decide the optimal hyper-parameters of SegTGAN, respectively. Then, the performances of SegTGAN on the Kits19 and Kits21 testing datasets are measured. The ACC, DSC, and SEN of SegTGAN on the Kits19 and Kits21 datasets are 0.9728/0.9526/0.9539 and 0.9676/0.9507/0.9344, respectively. Some segmentation results of SegTGAN on Kits21 are shown in Figure 4. 

SegTGAN is an enhanced generative adversarial segmentation model. In order to force the development of segmentation results that resemble the ground truth, the generator uses a dense block. To guarantee more accurate results, it is supplied to the discriminator along with the labels. To verify that the enhanced generative adversarial network framework can improve the segmentation performance, the performance of four network structures, namely U-Net, FCN, SegAN, and SegTGAN, are compared. The final metric results are obtained by computing each two-dimensional slice and averaging the results. 

### 3.1. Qualitative Evaluation 

**Figure 5:**
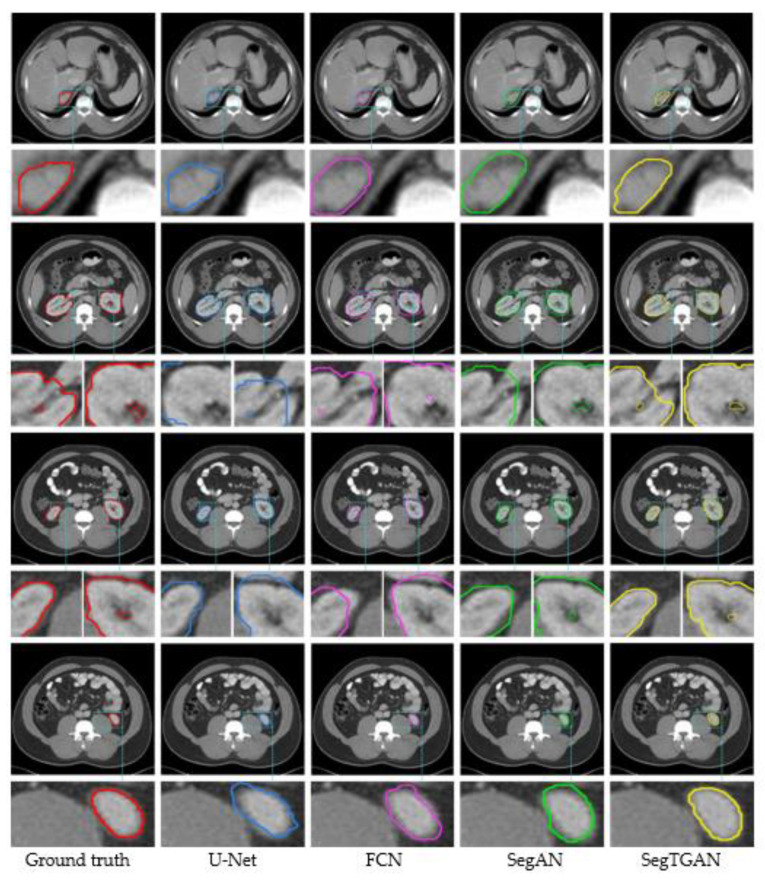
The segmentation results of each network in different slices on the Kits19 dataset. For the same CT slice, each row displays the kidney segmentation results from various networks. The truth labels are displayed in the first column, and the kidney contours produced using U-Net, FCN, SegAN, and SegTGAN, respectively, are displayed in columns 2 through 5, with local zooms below. The segmented kidney’s outline is the area that the colored curve has encircled.

**Figure 6:**
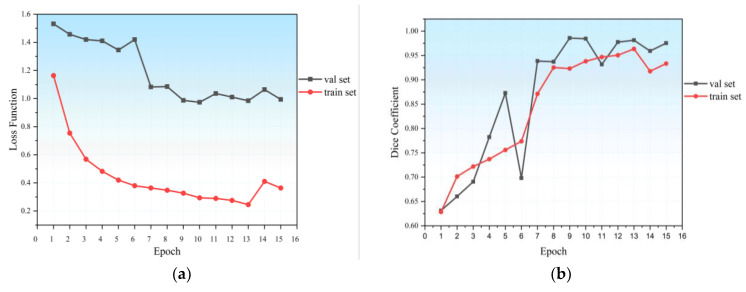
(**a**) Loss function of SegTGAN on Kits19; and (**b**) Dice coefficient of SegTGAN on Kits19.

The SegTGAN model and the additional neural networks U-Net, FCN, and SegAN were used to segment the experimental data, as shown in Figure 5. The segmentation results demonstrate the presence of kidneys in various slices, including the right kidney alone, the left kidney alone, and both the left and right kidneys. The fifth column in Figure 5 displays the segmentation outcomes that the SegTGAN model examined. In comparison to the other network segmentation outcomes, our model not only segments the outer contour well but also segments the inside hollow region with good results. Figure 6 depicts the changes in the loss function and Dice coefficients during training and validation. The loss functions of the training and validating sets steadily diminish over the model training period, and the Dice coefficients gradually rise as the number of training rounds rises. These trends imply that our SegTGAN algorithm may enhance kidney segmentation accuracy based on all the CT slices.

### 3.2. Quantitative Evaluation

As indicated in Table 2 and Table 3, the VOE, ASD, DSC, ACC, and SEN of U-Net are 0.1874/1.09/0.8968/0.9688/0.9146 and 0.2626/1.12/0.7522/0.9568/0.9296 on the Kits19 and Kits21 testing datasets, respectively. The results of FCN on the Kits19 and Kits21 testing datasets are 0.2101/0.87/0.8758/0.9693/0.8985 and 0.2521/1.09/0.8418/0.9564/0.9294, respectively. Moreover, the VOE, ASD, DSC, ACC, and SEN of the SegTGAN on the Kits19 and Kits21 testing datasets are 0.1617/0.61/0.9228/0.9728/0.9529 and 0.2260/1.03/0.9507/0.9301/0.9676/0.9344, respectively. The results of U-Net, FCN, and SegTGAN indicate that a single segmentation model cannot segment the kidney with ideal performance. Compared with the SegTGAN, the VOE, ASD, DSC, ACC, and SEN of SegAN on the Kits19 and Kits21 testing datasets are 0.1736/0.68/0.9014/0.9717/0.9250 and 0.2343/1.07/0.8960/0.9671/0.9268, respectively. The results verify that the introduction of a dense block structure into generative adversarial networks in this paper can improve the performance of the kidney segmentation task. To determine whether there was a statistically significant performance difference between our segmentation approach and the others, we performed a Wilcoxon signed rank test. Our model performs significantly better than the other models in relation to the majority of indicators (*p*-value < 0.05).

## 4. Discussion

In an abdominal CT scan, renal segmentation refers to the kidney organ’s complete marginal segmentation. The findings of the segmentation process demonstrate that some photos require additional analysis using more sophisticated methods. In this study, we developed a SegTGAN segmentation model and compared it to existing segmentation techniques. The adversarial nature of the network as a whole is what the model depends on. The generator processes the incoming CT picture and produces segmentation results that resemble real labels. These results are given to the discriminator at the same time as the labels. The discriminator network filters the input and produces a binary result that can be trained in an antagonistic way to the generator and, eventually, achieve equilibrium.

This research has several restrictions. Even though the method has a high accuracy, the accuracy is limited because the study employed a publicly available dataset and the practical situation has a low amount of data. In future work, we will further tune the network and test it on different datasets and clinical data to improve its robustness. Moreover, we will try to apply the model to the segmentation of MRI images. 

## 5. Conclusions

A novel deep neural network, namely SegTGAN, is proposed in this paper. The contributions of this work are as follows. First, in this model, the generator network is constructed using densely connected blocks and an encoder-decoder structure, while the discriminator network is constructed using a multi-scale convolutional network. Second, it is suggested that the corresponding loss functions for the two networks be used to optimize the objective function and boost the segmentation performance. In the kidney segmentation of CT scans from the Kits19 and Kits21 datasets, the segmentation results of SegTGAN are fairly close to the actual data (ground truth). Compared to U-Net, FCN, and SegAN, the DSC and SEN on the Kits19 testing dataset of SegTGAN are improved by 2.6%/3.93%, 4.7%/5.54%, and 2.14%/2.89%, respectively, while the DSC and SEN on the Kits21 testing dataset are improved of SegTGAN by 17.79%/0.48%, 8.83%/0.5%, and 3.41%/0.76%, respectively. In comparison to the other models, SegTGAN is more effective at segmenting medical images. The SegTGAN model can be evaluated for inclusion in practical applications because it is a more effective and reliable CT segmentation algorithm for kidneys when qualitative and quantitative characteristics are compared. 

## Figures and Tables

**Table 1:** Pros and cons of several representative methods.

Reference	Year	Method	Advantage	Disadvantage
Yan et al. [4]	2010	Connected component labeling algorithm and region growing approach	Leverage morphological features	Long and time consuming
Ronneberger et al. [17]	2015	U-Net	Multi-scale feature fusion	Prone to underfitting
Shelhamer et al. [16]	2017	FCN	Enable end-to-end segmentation	Poor detail in segmentation results
Pedraza et al. [19]	2017	AlexNet	First successful application of Trick such as ReLU, Dropout, and LRN in CNN	Increase in computational volume; redundancy of some feature information
Conze et al. [24]	2021	GAN	No need to design models that follow any kind of factorization	Non-convergence; collapse problem

**Table 2:** Comparison of U-Net, FCN, SegAN, and SegTGAN on the Kits19 dataset.

Model	VOE	ASD	DSC			ACC	SEN	SPE
		(mm)	Max	Min	Mean			
U-Net	18.74% ± 6.75%	1.09 ± 0.46	93.12%	54.23%	89.68% ± 4.30%	96.88%	91.46%	95.29%
FCN	21.01% ± 5.82%	0.87 ± 0.50	91.98%	48.11%	87.58% ± 7.54%	96.93%	89.85%	95.46%
SegAN	17.36% ± 2.43%	0.68 ± 0.20	94.72%	**63.16%**	90.14% ± 6.71%	97.17%	92.50%	95.54%
SegTGAN	**16.17% ± 2.13%**	**0.61 ± 0.17**	**95.26%**	58.30%	**92.28% ± 3.24%**	**97.28%**	**95.39%**	**96.12%**

**Table 3:** Comparison of U-Net, FCN, SegAN, and SegTGAN on the Kits21 dataset.

Model	VOE	ASD	DSC			ACC	SEN	SPE
		(mm)	Max	Min	Mean			
U-Net	26.26% ± 0.10%	1.12 ± 0.62	81.61%	50.30%	75.22% ± 5.01%	95.68%	92.96%	98.49%
FCN	25.21% ± 0.12%	1.09 ± 0.65	88.37%	55.12%	84.18% ± 3.93%	95.64%	92.94%	98.41%
SegAN	23.43% ± 0.10%	1.07 ± 0.57	92.30%	58.40%	89.60% ± 4.87%	96.71%	92.68%	98.58%
SegTGAN	**22.60%** **± 0.10%**	**1.03** **±** **0.52**	**95.07%**	**59.02%**	**93.01% ± 2.55%**	**96.76%**	**93.44%**	**98.62%**

## Data Availability

Not applicable.
